# Comparative Distribution of High-Risk Human Papillomavirus (HPV) Genotypes in India vs World Health Organization Regions and Countries: Implications for HPV Vaccination

**DOI:** 10.31486/toj.25.0082

**Published:** 2026

**Authors:** Anusree Prabhakaran, Srinivasan Vijayakumar, Nikitha S, Arathi P. Rao

**Affiliations:** ^1^ Department of Global Public Health Policy and Governance, Prasanna School of Public Health, Manipal Academy of Higher Education, Manipal, India; ^2^ Department of Radiology, Ochsner Clinic Foundation, New Orleans, LA; ^3^ Department of Radiation Oncology, Kasturba Medical College, Manipal Academy of Higher Education, Manipal, India; ^4^ Cancer Care, Cancer Care Advisors and Consultants LLC, Ridgeland, MS; ^5^ Cancer Control Program, Mississippi State Department of Health, Jackson, MS; ^6^ Manipal Institute of Virology, Manipal Academy of Higher Education, Manipal, India

**Keywords:** *Female*, *genotype*, *global health*, *human papillomavirus viruses*, *papillomavirus infections*, *papillomavirus vaccines*, *uterine cervical neoplasms*, *World Health Organization*

## Abstract

**Background:**

High-risk human papillomavirus (HPV)-induced cervical cancer is a major cause of mortality worldwide. Published data suggest geographic variations in the prevalence of some of the 12 high-risk HPV genotypes. Although the variations in prevalence of the 2 most common high-risk HPV genotypes—16 and 18—have been relatively well documented, the variations in prevalence of the other 10 high-risk genotypes are not as well documented. Reliable proof of variations in prevalence may warrant different high-risk HPV vaccine compositions/vaccination strategies in specific geographic areas. To explore the adequacy of the HPV vaccines available in India, we focused this study on the following: (1) confirming that high-risk HPV genotype variations exist on the Indian subcontinent, (2) determining how the genotype variations differ from the variations in other WHO regions/countries and among different regions/states in India, (3) determining whether any regional variations are sufficiently different to warrant changes in high-risk HPV vaccine compositions and/or vaccination strategies, and (4) determining whether the collected data could lead to new hypothesis generation and study design.

**Methods:**

We used 2 methods to gather information. To examine variations in prevalence, we conducted a secondary analysis (data mining) of publicly available data from the HPV Information Centre. The HPV Information Centre data were obtained from a systematic review of studies published from 1990 to 2015. We also conducted a scoping review of the literature published from 2015 to 2024.

**Results:**

As expected, we found variations in the prevalence of high-risk HPV genotypes among different regions on the Indian subcontinent. The predominant genotypes identified on the Indian subcontinent, in World Health Organization (WHO) regions, and in WHO South-East Asia Region countries were HPV 16 and HPV 18; other genotypes had varied prevalences. The scoping review of the literature revealed many inhomogeneities among the studies. None of the studies included testing for all 12 high-risk HPV genotypes, confidence intervals were rarely reported, and the quality assurance details of the studies are unknown.

**Conclusion:**

Although high-risk HPV genotype variations in prevalence were detected among different regions on the Indian subcontinent, the strength of the data does not justify different HPV vaccination compositions and strategies at this time. Studies are needed that include all 12 high-risk HPV genotypes, use uniform state-of-the-art high-risk HPV detection technologies, and document stringent quality assurance procedures. While our results must be interpreted with caution, our data can help inform future well-designed, homogenous, high-quality studies.

## INTRODUCTION

All over the world, 1 female dies every 2 minutes from cervical cancer.^1^ Globally, in 2022, approximately 662,301 females were newly diagnosed with cervical cancer, and 348,874 females died from cervical cancer.^2^ Most deaths occurred in lower- and middle-income countries,^3^ with 1 in 5 cervical cancer cases reported from India.^4^ Among Indian women, cervical cancer is the second-most common cancer^5^ with an age-standardized incidence of 17.7 cases per 100,000 women,^6^ more than twice the rate in the United States (6.3 cases per 100,000 women).^6^

Cervical cancer is principally caused by persistent infection with the human papillomavirus (HPV), which is transmitted through sexual contact. Twelve high-risk HPV genotypes cause cervical cancer—HPV 16, 18, 31, 33, 35, 39, 45, 51, 52, 56, 58, and 59—with HPV 16 and HPV 18 being the most common genotypes.^7^

Cervical cancer is highly preventable and treatable.^3^ In 2020, the World Health Organization (WHO) announced a global strategy to accelerate the elimination of cervical cancer. By 2030, the WHO aims for 90% of girls to be fully vaccinated against high-risk HPV by age 15, for 70% of women to be screened with a high-performance test by age 35 and again by age 45, and for 90% of women diagnosed with cervical disease to receive appropriate treatment.^1^

Vaccination against HPV is a vital prevention strategy,^1^ and India is taking steps to increase vaccination of girls aged 9 to 14 years.^8^ The HPV vaccines currently available in India are Cervarix, which safeguards from infection from HPV 16 and 18 (bivalent); Gardasil 4, which provides protection from HPV 6, 11, 16, and 18 (quadrivalent); GARDASIL 9, which provides protection from HPV 6, 11, 16, 18, 31, 33, 45, 52, and 58 (nonavalent)^9^; and CERVAVAC, introduced in India in January 2023, which protects against HPV 6, 11, 16, and 18 (quadrivalent).^10,11^ Although HPV 6 and 11 (genotypes for genital warts) are not considered high-risk for cancer, they are included in some vaccine compositions to prevent genital warts.

For HPV vaccination to be successful, the prevalence of the various HPV genotypes in the population must be known because intranational, international, and continental variations exist.^12,13^ A systematic review by Okoye et al identified the prevalent HPV genotypes in Africa as HPV 16, 52, 35, 18, and 58, whereas in Asia (India was not included in the study), the prevalent HPV genotypes were HPV 16, 52, 58, 33, and 18.^14^ Understanding regional variations is vital for tailoring vaccine compositions and vaccination strategies for specific populations to ensure that the vaccines are effective^13^ against all potential HPV genotypes in the region.

To achieve the WHO goal of eliminating cervical cancer, a detailed, region-specific map of the currently known 12 high-risk HPV genotypes and newly emerging minor high-risk HPV genotypes is needed. Such a map of variations in prevalence of high-risk HPV genotypes could help with fine-tuning vaccination compositions and strategies in different regions.

One objective for this retrospective data mining and scoping review of the literature study was to determine the variations in prevalence of high-risk HPV genotypes on the Indian subcontinent and to examine how the overall Indian prevalence differs from the prevalence in other global regions/countries and within different regions/states in India. Another objective was to ascertain the reliability of the data. We defined *reliable data* as data and studies that at a minimum included all 12 genotypes of high-risk HPV in the determination of variations in prevalence. If reliable data about regional variations exist, the information could be used to identify the need for different high-risk HPV vaccine compositions and/or vaccination strategies in various areas of India. However, if reliable data show no significant intranational or international variations in the prevalence of high-risk HPV genotypes, such a finding could help prevent the unnecessary expenditure of resources on research, clinical trials, and HPV vaccine genotype composition; resources could be redirected to strategic cervical cancer elimination plans. On the other hand, if the data are not reliable enough to support substantive conclusions and recommendations, the information could potentially lead to new hypothesis generation and inform the design of new studies.

## METHODS

We used 2 methods to obtain the full scope of the data. The first methodology was data mining from the HPV Information Centre database. These results include specific WHO regions, India, and countries in the WHO South-East Asia Region. The second methodology was a scoping review of the literature that was limited to India for the period 2015 to 2024. We did not combine HPV Information Centre data with the findings of our scoping review of the literature for any pooled prevalence estimates. No statistical modeling or meta-analysis was performed, and all findings are presented descriptively.

### HPV Information Centre Data Mining

After obtaining formal permission from the HPV Information Centre, we conducted a secondary analysis (data mining) of publicly available data from the HPV Information Centre, a collaboration between the International Agency for Research on Cancer (IARC) and the Catalan Institute of Oncology (ICO).^15^ For our analysis, we used the latest full report (published in 2023) of the different regions and countries. The HPV Information Centre data are based on a systematic review of studies published between 1990 and 2015 that assess global and regional HPV prevalence and type distribution for cervical cancer, low-grade and high-grade cervical lesions, and normal cytology. Included studies had at least 20 cases of carcinoma and lesions and 100 cases of normal cytology.^15,16^ Data were pooled globally and regionally, with 95% binomial confidence intervals (CIs) calculated for HPV prevalence. The methodology used by IARC/ICO is detailed in Bruni et al.^16^

The HPV Information Centre provides HPV-related statistics for almost all countries, but data are categorized by continents and countries instead of by WHO regions. We obtained HPV Information Centre data for the continents of Africa, America, and Europe. Because the HPV Information Centre does not provide data for the WHO Western Pacific Region, Eastern Mediterranean Region, or South-East Asia Region, we did not include these regions in our study. Instead, we accessed the latest full report (published in 2023) for India, along with reports from 4 South-East Asia Region countries (Bhutan, Indonesia, Nepal, and Thailand) from the HPV Information Centre website.^15^ Even though Pakistan is not included in the WHO South-East Asia Region, we included Pakistan in our analysis because of the country's geographic proximity to countries in the South-East Asia Region, especially India. Data were not available for other countries in the WHO South-East Asia Region, such as Bangladesh, Democratic People's Republic of Korea, Maldives, Myanmar, Sri Lanka, and Timor-Leste, so these countries are not included in our analysis.

### Scoping Review of the Literature

Because the HPV Information Centre data period ends with June 30, 2015, we conducted a scoping review of the literature on the prevalence of high-risk HPV genotypes in India from 2015 to 2024. We searched the literature and used the Preferred Reporting Items for Systematic reviews and Meta-Analyses extension for Scoping Reviews (PRISMA-ScR) checklist to report the results of the review.^17^ We followed the Arksey and O’Malley framework for scoping reviews.^18^

We chose the population, concept, and context framework to identify relevant literature to determine the prevalence of high-risk HPV genotypes in India and to determine if regional variation exists in the prevalence of the high-risk HPV genotypes:

- *Population.* Studies conducted on women with normal cervical cytology, women with low-grade and high-grade precancerous cervical lesions, and women with cervical cancer were included in the review.
- *Concept.* Studies on the prevalence of any of the 12 high-risk HPV genotypes were included in the review.
- *Context.* Studies conducted only in Indian states were included in the review.

We created a search string using relevant keywords from the Medical Subject Headings library. The search strategy was validated with subject experts (SV, APR, NS). PubMed, Web of Science, Embase, and Google Scholar were searched on May 12, 2024. The search string was translated from PubMed to other databases using a polyglot search translator. Studies from 2015 to 2024 were considered for this review. Appendix 1 provides the detailed search strategy.

We retrieved a total of 9,697 studies: PubMed (n=2,150), Embase (n=4,505), Web of Science (n=2,956), and Google Scholar (n=86). Search results were imported into the Rayyan software (Rayyan Systems, Inc).^19^ After 4,178 duplicate studies were removed, the filters “India,” “Indian,” “Asia,” and “Asian” were applied to restrict the results to studies from India, leaving 278 papers. Two reviewers (AP and SV) screened the titles and abstracts of these studies for applicability. Disagreements were resolved after consultation with a third reviewer (APR). Thirty-six studies were determined to be appropriate for full-text screening. Full text could not be retrieved for 8 of 36 studies, so reviewer SV screened the full text of 28 studies. Eighteen studies were excluded for the following reasons: 7 studies were conducted with patients who had cervical cancer or HIV (wrong population), 6 studies did not include HPV genotypes (wrong outcome), 2 studies were not conducted in India (wrong study setting), 1 study was a duplicate report, and 2 studies had small sample sizes. Ten studies were deemed acceptable for the scoping review. We also conducted a citation search of the included studies, and 1 additional study was identified from the reference list of an article.

**Figure 1.**
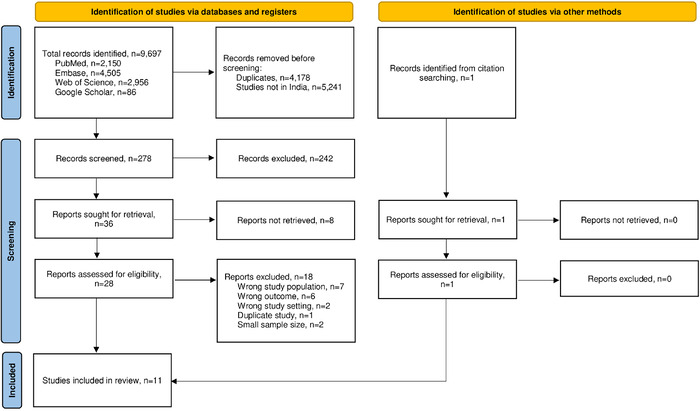
**Preferred Reporting Items for Systematic reviews and Meta-Analyses (PRISMA) flow diagram shows the study selection for the scoping review of the literature.** One study was a duplicate of an included study but had a different title and authors. We included the most recently published study^30^ in the review and excluded the other study.

Eleven studies were included in the scoping review of the literature.^20-30^ The PRISMA flow chart is presented in Figure 1. A list of the 18 studies excluded during full-text screening is provided in Appendix 2.

For the 11 studies included in the review, we charted the following data on a predesigned data extraction table: citation details, region where the study was conducted, type of technique used for HPV detection, type of cervical lesions, and the prevalence of the 12 high-risk HPV genotypes. Data are summarized narratively and supported by tables. Because the goal of the scoping review of the literature was to outline the prevalence of high-risk HPV genotypes in India, risk of bias assessments and quality assessments of the included studies were not conducted.

## RESULTS

### HPV Information Centre Data From Published Studies, 1990 to 2015

**Figure 2.**
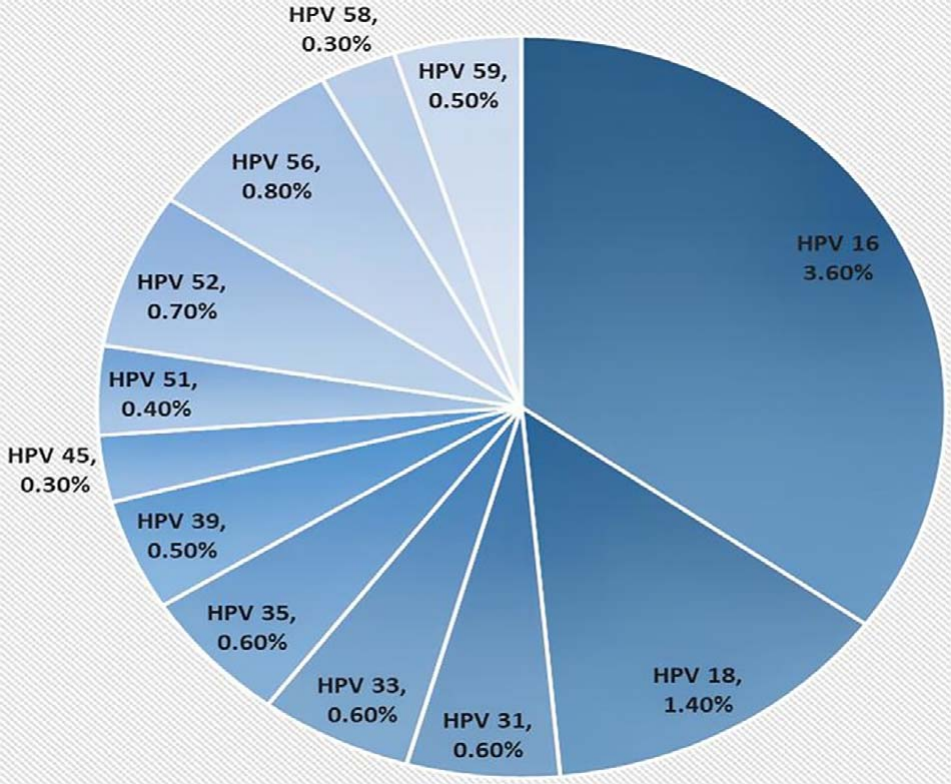
**The pooled prevalence percentages for 12 high-risk human papillomavirus (HPV) genotypes among females with normal cervical cytology in India.** Crude age-specific HPV prevalence percentages and 95% confidence intervals in females with normal cervical cytology in India are provided in Appendix 3.

Figure 2 shows the pooled prevalence percentages for 12 high-risk HPV genotypes in females with normal cervical cytology in India, and Appendix 3 shows HPV prevalence percentages with 95% CIs by age group. A comparison of the pooled prevalences of 12 high-risk HPV genotypes in India vs the WHO regions of Africa, America, and Europe is presented in Table 1 with binomial 95% CIs for each HPV genotype.

**Table 1.** Pooled Prevalences of High-Risk Human Papillomavirus (HPV) Genotypes in India vs Three World Health Organization Regions

HPV Genotype	India	Africa	India vs Africa	America	India vs America	Europe	India vs Europe
16	3.6 (3.2-4.0) n=8,845	2.4 (2.2-2.6) n=19,726	+1.2	3.3 (3.2-3.4) n=105,042	+0.3	2.8 (2.7-2.8) n=180,090	+0.8
18	1.4 (1.1-1.6) n=8,845	1.4 (1.2-1.5) n=19,726	0	1.2 (1.1-1.3) n=104,589	+0.2	1.0 (0.9-1.0) n=178,318	+0.4
31	0.6 (0.4-1.0) n=2,366	1.1 (0.9-1.2) n=19,420	–0.5	1.3 (1.2-1.3) n=101,222	–0.7	1.5 (1.4-1.6) n=164,983	–0.9
33	0.6 (0.4-1.0) n=2,366	0.9 (0.8-1.0) n=19,420	–0.3	0.6 (0.6-0.7) n=101,346	0	0.7 (0.7-0.7) n=164,623	–0.1
35	0.6 (0.4-1.0) n=2,366	1.7 (1.5-1.9) n=19,324	–1.1	0.6 (0.5-0.6) n=100,087	0	0.4 (0.3-0.4) n=153,819	+0.2
39	0.5 (0.3-0.9) n=2,366	0.7 (0.6-0.8) n=18,288	–0.2	1.1 (1.0-1.2) n=99,690	–0.6	0.9 (0.8-0.9) n=152,061	–0.4
45	0.3 (0.1-0.6) n=2,366	1.3 (1.2-1.5) n=19,324	–1	0.8 (0.8-0.9) n=100,495	–0.5	0.7 (0.7-0.8) n=155,359	–0.4
51	0.4 (0.2-0.7) n=2,366	1.1 (1.0-1.3) n=18,288	–0.7	1.3 (1.2-1.4) n=95,789	–0.9	1.3 (1.2-1.4) n=154,244	–0.9
52	0.7 (0.4-1.1) n=2,366	1.7 (1.6-1.9) n=18,384	–1	1.2 (1.2-1.3) n=99,941	–0.5	1.2 (1.1-1.2) n=153,700	–0.5
56	0.8 (0.5-1.3) n=2,366	0.9 (0.7-1.0) n=18,288	–0.1	0.8 (0.8-0.9) n=99,878	0	0.8 (0.8-0.9) n=153,487	0
58	0.3 (0.1-0.6) n=2,366	1.7 (1.5-1.9) n=18,384	–1.4	1.0 (1.0-1.1) n=99,215	–0.7	0.7 (0.6-0.7) n=155,224	–0.4
59	0.5 (0.3-0.9) n=2,366	0.8 (0.6-0.9) n=18,288	–0.3	1.1 (1.0-1.1) n=100,053	–0.6	0.7 (0.6-0.7) n=150,513	–0.2

Note: India and World Health Organization region prevalence data are shown as percentages with 95% confidence intervals.

**Figure 3.**
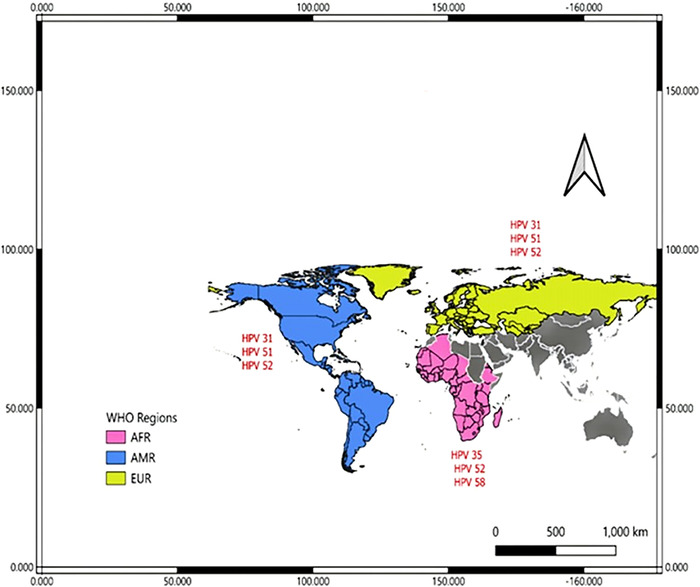
**Dominant high-risk human papillomavirus (HPV) genotypes other than HPV 16 and HPV 18 in the 3 World Health Organization regions of Africa (AFR), America (AMR), and Europe (EUR) from the HPV Information Centre database.** The pooled prevalences data are based on a systematic review of studies conducted between 1990 and 2015. The HPV Information Centre database does not include data for the World Health Organization Western Pacific Region, Eastern Mediterranean Region, or South-East Asia Region, so data from these regions are not included.

Compared to Africa, America, and Europe, India had a higher prevalence of HPV 16 by 1.2%, 0.3%, and 0.8%, respectively. The prevalence of HPV 18 was the same in India and Africa but 0.2% and 0.4% higher in India compared to America and Europe. However, prevalences of the other high-risk HPV genotypes were comparatively less prevalent in India than in Africa, America, and Europe. For example, the prevalence of HPV 31 was 0.6% in India compared to 1.1% in Africa, 1.3% in America, and 1.5% in Europe. Similarly, the prevalence of HPV 45 was 0.3% in India compared to 1.3% in Africa, 0.8% in America, and 0.7% in Europe. Figure 3 shows the dominant high-risk HPV genotypes other than HPV 16 and HPV 18 in the WHO regions of Africa, America, and Europe.

A comparison of the pooled prevalences of 12 high-risk HPV genotypes in India vs Pakistan and 4 countries in the WHO South-East Asia Region—Bhutan, Indonesia, Nepal, and Thailand—is presented in Table 2 with binomial 95% CIs for each HPV genotype.

**Table 2.** Pooled Prevalences of High-Risk Human Papillomavirus (HPV) Genotypes in India vs Pakistan and Four Countries in the World Health Organization South-East Asia Region

HPV Genotype	India	Bhutan	India vs Bhutan	Indonesia	India vs Indonesia	Nepal	India vs Nepal	Thailand	India vs Thailand	Pakistan	India vs Pakistan
16	3.6 (3.2-4.0) n=8,845	3.4 (2.8-4.3) n=2,272	+0.2	2.0 (0.8-5.0) n=200	+1.6	1.4 (0.8-2.2) n=1,109	+2.2	2.1 (1.8-2.5) n=5,693	+1.5	0.5 (0.2-1.2) n=877	+3.1
18	1.4 (1.1-1.6) n=8,845	2.3 (1.8-3.0) n=2,272	–0.9	2.0 (0.8-5.0) n=200	–0.6	0.6 (0.2-1.3) n=898	+0.8	1.3 (1.1-1.7) n=5,533	+0.1	0.0 (0.0-0.4) n=877	+1.4
31	0.6 (0.4-1.0) n=2,366	0.9 (0.6-1.4) n=2,272	–0.3	0.0 (0.0-1.9) n=200	+0.6	0.1 (0.0-0.6) n=898	+0.5	1.1 (0.8-1.6) n=2,798	–0.5	NA	NA
33	0.6 (0.4-1.0) n=2,366	0.9 (0.6-1.4) n=2,272	–0.3	0.0 (0.0-1.9) n=200	+0.6	0.2 (0.1-0.8) n=898	+0.4	1.6 (1.2-2.2) n=2,798	–1	0.0 (0.0-0.4) n=877	+0.6
35	0.6 (0.4-1.0) n=2,366	0.4 (0.2-0.8) n=2,272	+0.2	0.0 (0.0-1.9) n=200	+0.6	0.3 (0.1-1.0) n=898	+0.3	0.2 (0.1-0.5) n=2,573	+0.4	0.2 (0.1-0.8) n=877	+0.4
39	0.5 (0.3-0.9) n=2,366	0.7 (0.4-1.1) n=2,272	–0.2	0.5 (0.1-2.8) n=200	0	0.4 (0.2-1.1) n=898	+0.1	0.2 (0.1-0.5) n=2,371	+0.3	NA	NA
45	0.3 (0.1-0.6) n=2,366	1.2 (0.9-1.8) n=2,272	–0.9	0.5 (0.1-2.8) n=200	–0.2	0.2 (0.1-0.8) n=898	+0.1	0.4 (0.2-0.7) n=2,371	–0.1	0.0 (0.0-0.4) n=877	+0.3
51	0.4 (0.2-0.7) n=2,366	1.3 (0.9-1.8) n=2,272	–0.9	4.5 (2.4-8.3) n=200	–4.1	0.2 (0.1-0.8) n=898	+0.2	0.2 (0.1-0.4) n=2,371	+0.2	0.2 (0.1-0.8) n=877	+0.2
52	0.7 (0.4-1.1) n=2,366	1.2 (0.9-1.8) n=2,272	–0.5	1.0 (0.3-3.6) n=200	–0.3	0.4 (0.2-1.1) n=898	+0.3	0.2 (0.1-0.5) n=2,471	+0.5	NA	NA
56	0.8 (0.5-1.3) n=2,366	1.4 (1.0-1.9) n=2,272	–0.6	2.0 (0.8-5.0) n=200	–1.2	0.7 (0.3-1.4) n=898	+0.1	0.2 (0.1-0.5) n=2,231	+0.6	0.2 (0.1-0.8) n=877	+0.6
58	0.3 (0.1-0.6) n=2,366	1.2 (0.8-1.7) n=2,272	–0.9	0.0 (0.0-1.9) n=200	+0.3	0.9 (0.5-1.7) n=898	–0.6	1.1 (0.8-1.6) n=2,471	–0.8	NA	NA
59	0.5 (0.3-0.9) n=2,366	1.8 (1.3-2.4) n=2,272	–1.3	0.5 (0.1-2.8) n=200	0	0.0 (0.0-0.4) n=898	+0.5	0.0 (0.0-0.3) n=2231	+0.5	0.1 (0.0-0.6) n=877	+0.4

Note: India, Pakistan, and World Health Organization South-East Asia Region country prevalence data are shown as percentages with 95% confidence intervals.

NA, not available.

Across India and the 5 countries, HPV 16 was the most prevalent genotype, with India having the highest prevalence at 3.6%. The prevalence in India was slightly higher than Bhutan (by 0.2%) and much higher than Indonesia (+1.6%), Nepal (+2.2%), Thailand (+1.5%), and Pakistan (+3.1%). For HPV 18, India had a lower prevalence than Bhutan and Indonesia but a higher prevalence than Nepal, Thailand, and Pakistan. The prevalences of HPV 31 and HPV 33 (both 0.6% in India) were less prevalent in India vs Bhutan and Thailand, with Indonesia and Pakistan having very low or no recorded prevalence of these 2 genotypes.

India and Indonesia had the same prevalence of HPV 39 (0.5%) which was 0.1% and 0.3% higher than Nepal and Thailand and 0.2% lower than Bhutan. The prevalence of HPV 45 in India was 0.9% lower than Bhutan, 0.2% lower than Indonesia, and 0.1% lower than Thailand but 0.1% and 0.3% higher than Nepal and Pakistan, respectively. The prevalence of HPV 51 was much lower in India compared to Indonesia (by 4.1%) and Bhutan (by 0.9%) but was 0.2% higher than Nepal, Thailand, and Pakistan. India had a slightly lower prevalence of HPV 52, 56, and 58 than Bhutan, while the prevalence of HPV 59 in India was 1.3% lower than Bhutan but higher than Nepal, Thailand, and Pakistan.

### Scoping Review of the Literature, 2015 to 2024

The 11 heterogeneous studies included in the scoping review of the literature were conducted in different geographic regions of India, and sample sizes varied widely, ranging from 217 to 2,278 participants.^20-30^ Although polymerase chain reaction (PCR)-based assays were the most common method for HPV detection, the studies differed in the specific platforms and protocols employed for HPV detection. None of the studies assessed the prevalence of all 12 high-risk HPV genotypes. Some studies did not provide CIs for prevalence values or detailed methodology descriptions, and not all studies provided the *n* for each genotype. Characteristics of the included studies are presented in Table 3.

**Table 3.** Characteristics of the Studies Included in the Scoping Review of the Literature

Study	Setting	Ethics Committee Approval	Sample Size	Age Group, Years	Population	Objective	Technique for HPV Detection^a^	Study Limitations
Sharma et al, 2015^21^	Central India (Madhya Pradesh, Jharkhand, Chhattisgarh)	Yes	2,278	9-25	Healthy tribal girls	To examine the status of HPV infection and genotype distribution in preadolescents (9-12 years), adolescents (13-17 years), and young adult girls (18-25 years) from 3 tribal states	Genomic DNA extraction + PCR	Not given
Peedicayil et al, 2016^29^	South India (Tamil Nadu)	Yes	809	≤50	Married women	To determine the community prevalence of HPV infection and the feasibility of self-collected vaginal swabs for HPV testing	Line blot assay	Study was part of a large screening project, and HPV results were not correlated with biopsy results. Samples were collected 10 years prior, and HPV epidemiology could change over time.
Senapati et al, 2017^26^	Eastern India (Odisha)	Yes	607	≥18	Women with symptoms of cervical cancer	To determine the genotypes, prevalence, and associated risk factors among women with and without cervical cancer	L1 PCR, sequencing, and E6/E7 nested multiplex type-specific PCR	HIV status and other diseases could not be assessed, which could influence the prevalence of HPV infection.
Ghosh et al, 2019^25^	South India (Karnataka)	Yes	1,140 tribal 1,100 general population	20-65	Tribal and general population women	To determine and compare the prevalence and risk factors of cervical viral infections among the tribal and general populations	PCR	Not given
Subramanian et al, 2021^24^	South India (Tamil Nadu)	Not given	1,523	<60	Women undergoing cervical cancer screening	To investigate the prevalence, genotype distribution, epidemiologic determinants, and dynamics of cervical HPV infection	Hybrid Capture 2 assay and linear array HPV genotyping	<40% of high-risk HPV–positive women from baseline screening were available for follow-up at 36 months.
Gupta et al, 2021^22^	Central India (Madhya Pradesh)	Yes	782	≥15	Married women	To evaluate HPV prevalence and describe high-risk HPV 16 distribution in the rural population	PCR with MY09/11 primers + genotyping	Not given
Kulkarni et al, 2023^20^	Central India (Indore, Madhya Pradesh)	Yes	736	21-60	High-risk women	To assess the prevalence of high-risk HPV transmission and the VIA positivity rate in females at greater risk of contracting HPV	VIA and RT-PCR	Not mentioned
Vora et al, 2023^28^	Northwest India (Ahmedabad, Gujarat)	Yes	1,714	30-45	Married women	To determine the prevalence of HPV in the urban population and the distribution of high-risk HPV genotypes in marginalized populations in urban slums	DNA-based method	Only slightly more than half of recruits underwent screening, and prevalence is based on number screened.
Bhattacharya et al, 2024^27^	Northeast India (Tripura)	Not given	499	18-80	Married women	To determine overall HPV prevalence, age-specific prevalence, and distribution of high-risk HPV genotypes in cervical samples	PCR with MY09/11 and GP5+/GP6+ primers	Not given
Punia and Sharma, 2024^30^	Northwest India (Rajasthan)	Yes	217	21-50	Women with no history of malignancy/pregnancy	To find the distribution and estimate the prevalence of high-risk HPV genotypes and correlate HPV DNA testing with Pap smears	Pap smear and HPV DNA testing	Only women attending the outpatient department at a tertiary care center were included.
Pankaj et al, 2024^23^	North India (Bihar)	Yes	1,510	25-75	Married women	To analyze the prevalence, risk factors, and genotype distribution of HPV infections among women at a tertiary care hospital	TaqMan-based RT-PCR	Not given

^a^Each study used a different technique.

DNA, deoxyribonucleic acid; HIV, human immunodeficiency virus; HPV, human papillomavirus; L1 PCR, L1 protein polymerase chain reaction; PCR, polymerase chain reaction; RT-PCR, real-time polymerase chain reaction; VIA, visual inspection with acetic acid.

Table 4 presents the prevalences of the 12 high-risk HPV genotypes by study. The most prevalent genotypes were HPV 16 and HPV 18, but prevalence varied across geographic areas.

**Table 4.** Prevalence of 12 High-Risk Human Papillomavirus (HPV) Genotypes From the Studies Included in the Scoping Review of the Literature^a^

				High-Risk Human Papillomavirus (HPV) Genotype Prevalence, %											
Study	Setting	Sample Size	Age Group, Years	HPV 16	HPV 18	HPV 31	HPV 33	HPV 35	HPV 39	HPV 45	HPV 51	HPV 52	HPV 56	HPV 58	HPV 59
Sharma et al, 2015^21^	Central India (Madhya Pradesh, Jharkhand, Chhattisgarh)	2,278 recruited; 2,034 samples eligible for analysis; 262 (12.9%) positive^b^	9-25	50.4	6.1	1.9	NA	NA	NA	NA	2.7	NA	NA	NA	NA
Peedicayil et al, 2016^29^^c^	South India (Tamil Nadu)	809 recruited; 76 (9.4%) positive	≤50	3	0.4	0.3	0.3	NA	0.3	0.4	NA	0.6	0.5	NA	0.1
Senapati et al, 2017^26^^d^	Eastern India (Odisha)	607 recruited; 595 samples eligible for analysis; 359(60.3%) positive; 346 samples processed for genotyping^e^	≥18	87.28	24.56	NA	NA	1.7	3.17	1.7	3.46	0.57	NA	1.1	NA
Ghosh et al, 2019^25^	South India (Karnataka)	1,140 tribal women recruited;	20-65	10.7	28.3	NA	NA	NA	NA	22.8	NA	NA	NA	NA	NA
		1,100 general population women recruited		9.1	4.1	NA	NA	NA	NA	8.1	NA	NA	NA	NA	NA
Subramanian et al, 2021^24^	South India (Tamil Nadu)	1,523 recruited; 193 (12.7%) positive; 178 samples eligible for analysis; 168 (94.3%) had single HPV infection^f^	Not given	27.3	3	NA	NA	5.3	NA	NA	NA	22.6	NA	14	3
Gupta et al, 2021^22^	Central India (Madhya Pradesh)	782 recruited; 56 (7.1%) positive^g^	≥15	95	NA	NA	NA	NA	NA	NA	NA	NA	NA	NA	NA
Kulkarni et al, 2023^20^	Central India (Indore, Madhya Pradesh)	736 recruited; 54 (7.3%) positive^h^	21-60	29.6	11.1	12.9	NA	NA	NA	9.2	NA	NA	NA	NA	NA
Vora et al, 2023^28^	Northwest India (Ahmedabad, Gujarat)	1,714 recruited; 956 screened; 248 positive^i^	30-45	53	31	1.9	1.4	NA	NA	4.2	NA	0.5	0.9	6.9	0.9
Bhattacharya et al, 2024^27^	Northeast India (Tripura)	499 recruited; 236 samples eligible for analysis; 214 (90.68%) positive^j^	18-80	53.27	7.01	NA	NA	NA	NA	NA	NA	NA	NA	NA	NA
Punia and Sharma, 2024^30^	Northwest India (Rajasthan)	217 recruited; 12 (5.5%) positive^k^	21-50	NA	16.7	NA	16.7	NA	NA	NA	16.7	NA	16.7	NA	33.3
Pankaj et al, 2024^23^	North India (Bihar)	1,510 recruited; 1,439 samples available for analysis; 537 (37.3%) positive^l^	25-75	76.5	10.2	2.4	3.2	0.7	0.6	1.3	NA	0.7	0.9	0.9	0.4

^a^Only 3 of 11 studies included at least 9 of the 12 high-risk HPV genotypes in the determination of the variation in prevalence of high-risk HPV. For that reason, only those 3 studies can be considered close to reliable.^23,28,29^ None of the studies included all 12 genotypes.

^b^The denominator used to calculate the prevalence of different HPV genotypes is 262.

^c^This study reported prevalence percentages of HPV in the cervix and the vagina. We considered the prevalence of HPV in the cervix.

^d^This study provided high-risk HPV prevalence in women with different cytologic results. We considered the combined prevalence of high-risk HPV.

^e^The denominator used to calculate the prevalence of different HPV genotypes is 346.

^f^The denominator used to calculate the prevalence of different HPV genotypes is 168.

^g^The denominator used to calculate the prevalence of different HPV genotypes is 56.

^h^The denominator used to calculate the prevalence of different HPV genotypes is 54.

^i^The denominator used to calculate the prevalence of different HPV genotypes is 248.

^j^The denominator used to calculate the prevalence of different HPV genotypes is 214.

^k^The denominator used to calculate the prevalence of different HPV genotypes is 12.

^l^The denominator used to calculate the prevalence of different HPV genotypes is 537.

NA, not analyzed.

In central India, Kulkarni et al (2023) reported an HPV 16 prevalence of 29.6% (n=16) in 54 high-risk HPV–positive women.^20^ In contrast, Sharma et al (2015) reported an HPV 16 prevalence of 50.4% (132/262) in healthy tribal girls from the central Indian states of Madhya Pradesh, Jharkhand, and Chhattisgarh.^21^ Gupta et al (2021) reported the much higher HPV 16 prevalence of 95% (53/56) among women who tested positive for HPV in Madhya Pradesh.^22^

In northern India, Pankaj et al (2024) reported an HPV 16 prevalence of 76.5% (411/537) among married, HPV-positive women in Bihar.^23^ In southern India, Subramanian et al (2021) reported an HPV 16 prevalence of 27.3% (46/168) in women with single HPV infection in Tamil Nadu,^24^ while Ghosh et al (2019) reported HPV 16 prevalences of 10.7% in 1,140 tribal women and 9.1% in 1,100 women from the general population in Karnataka.^25^ In eastern India (Odisha), Senapati et al (2017) reported a much higher HPV 16 prevalence of 87.28% (302/346).^26^ In northeast India, Bhattacharya et al (2024) reported a high HPV 16 prevalence of 53.27% (114/214) in married, HPV-positive women in Tripura.^27^

Infection with HPV 18 also exhibited regional variations. The reported prevalence of HPV 18 was highest in northwest India (Ahmedabad, Gujarat), with a rate of 31% in 248 women.^28^ A similar prevalence of 28.3% in 1,140 tribal women was reported from south India (Karnataka).^25^ In eastern India (Odisha), the prevalence was 24.56% (85/346).^26^ In contrast, the prevalence in Tamil Nadu was much lower: 0.4% in 809 women^29^ and 3% in 178 women.^24^

Prevalence rates for other high-risk HPV types—HPV 31, 33, 35, 39, 45, 51, 52, 56, 58, and 59—were reported in some studies. However, as previously noted, none of the studies investigated all 12 genotypes. Kulkarni et al reported that HPV 31 had a slightly higher prevalence (12.9% [7/54]) than HPV 18 (11.1% [6/54]) in Central India, and HPV 45 had a similar prevalence of (9.2% [5/54]).^20^ Sharma et al reported prevalences of 2.7% (7/262) for HPV 51 and 1.9% (5/262) for HPV 31 among women in central India (Madhya Pradesh, Jharkhand, and Chhattisgarh).^21^

After HPV 16 and HPV 18, the highly prevalent genotypes were HPV 33 (3.2% [17/537]), followed by HPV 31 (2.4% [13/537]) and HPV 45 (1.3% [7/537]) in north India.^23^ In northwest India, Punia and Sharma (2024) found HPV 59 to have the highest prevalence of all the genotypes in Rajasthan at 33.3% (n=12), followed by HPV 18, 33, 51, and 56, with each having a prevalence of 16.7% (n=12),^30^ while in Ahmedabad, Gujarat, Vora et al (2023) recorded HPV 58 (6.9%) and HPV 45 (4.2%) as the most frequently detected types (n=248).^28^ In Odisha in eastern India, Senapati et al reported the following prevalences: 1.7% for HPV 35 (6/346), 3.17% for HPV 39 (11/346), 1.7% for HPV 45 (6/346), 3.46% for HPV 51 (12/346), 0.57 % for HPV 52 (2/346), and 1.1% for HPV 58 (4/346).^26^

Two studies conducted in Tamil Nadu in southern India reported variations in genotype distribution. Peedicayil et al (2016) reported that after HPV 16 and HPV 18, the prevalent genotypes were HPV 52 (0.6%), HPV 56 (0.5%), and HPV 45 (0.4%) (n=809).^29^ Subramanian et al reported a much higher HPV 52 prevalence at 22.6% (n=178).^24^

**Figure 4.**
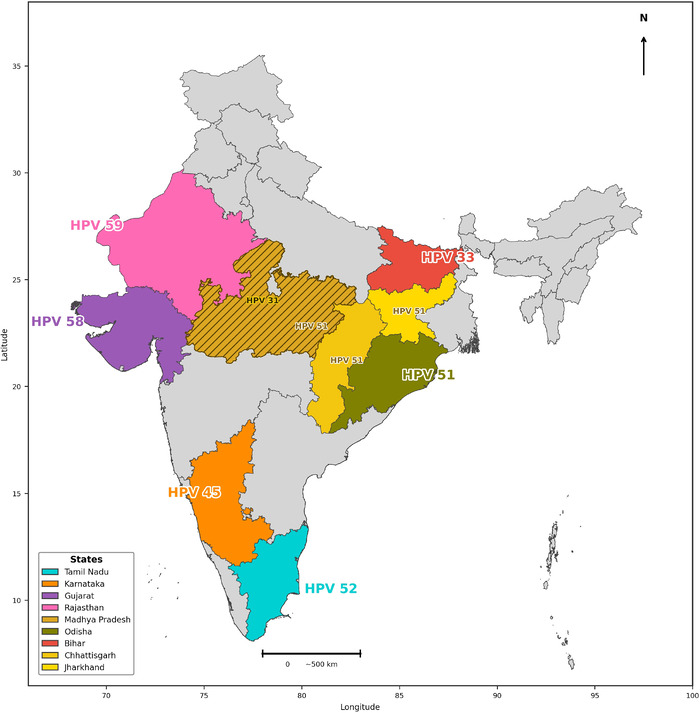
**Most common high-risk human papillomavirus (HPV) genotypes other than HPV 16 and HPV 18 in India as determined from the scoping review of the literature (2015 to 2024).** Related references are as follows: Tamil Nadu,^24,29^ Karnataka,^25^ Gujarat,^28^ Rajasthan,^30^ Madhya Pradesh,^20,21^ Odisha,^26^ Bihar,^23^ and Chhattisgarh and Jharkhand.^21^ Note for the Madhya Pradesh, Chhattisgarh, and Jharkhand region, HPV 31 is specific to Madhya Pradesh^20^ and HPV 51 is common to Madhya Pradesh, Chhattisgarh, and Jharkhand.^21^

Overall, these studies show that HPV 16 and HPV 18 are the most common genotypes in India, but the data demonstrate regional differences in the distribution of high-risk HPV genotypes beyond HPV 16 and HPV 18, with HPV 33, 45, 51, 52, 58, and 59 the most frequently detected types (Figure 4).

## DISCUSSION

Figures 2, 3, and 4 and Tables 1 through 4 show the variations in prevalence of high-risk HPV genotypes in selected WHO regions, in countries from the WHO South-East Asia Region and Pakistan, and in different regions in India. HPV 16 and HPV 18 are the predominant high-risk HPV subtypes in the WHO regions of Africa, America, and Europe; in Pakistan and the different countries of the South-East Asia Region; and in the different regions of India.

The prevalence of HPV 16 is higher on the Indian subcontinent than in Africa, America, and Europe, and the prevalence of HPV 18 is higher on the Indian subcontinent than in America and Europe but similar to Africa (1.4%). As shown in Table 1, the prevalences of HPV 31, 33, 35, 39, 45, 51, 52, 56, 58, and 59 are higher in Africa vs India. The prevalences of HPV 31, 33, 35, 39, 45, 51, 52, 56, 58, and 59 in India are less than or equal to the prevalences in America. In the comparison with Europe, the prevalence comparison for these high-risk genotypes is more complex, with some genotypes showing a higher prevalence in India, some showing a lower prevalence in India, and one genotype showing no difference.

India had a higher prevalence of HPV 16 than Pakistan and the 4 countries in the WHO South-East Asia Region analyzed in Table 2. The prevalence of HPV 18 in India was lower than Bhutan and Indonesia but higher than Nepal, Thailand, and Pakistan. For the other HPV genotypes, no consistent trends could be discerned between India and the 5 other countries.

On the Indian subcontinent, although regional variations were seen,^20-30^ the most prevalent HPV genotypes were HPV 16 and HPV 18. HPV 16 was the predominant genotype, followed by HPV 18, with the exceptions of Karnataka where HPV 18 was more prevalent than HPV 16 in the tribal population^25^ and the state of Rajasthan where the most prevalent genotype was HPV 59 (33%).^30^

Other than HPV 16 and HPV 18, we found no consistency among studies in the methodology used to include other genotypes for determination of the variation in prevalence of high-risk HPV. No definitive conclusions can be drawn from the data because none of the studies included all 12 genotypes in the determination of the variation in prevalence of high-risk HPV, quality control measures were not consistently reported (data not shown in the tables), and different techniques were used for HPV detection.

With some limitations, we achieved the objectives of this study: determining the variations in prevalence of high-risk HPV genotypes on the Indian subcontinent, examining how the overall Indian prevalence and the prevalence in different regions/states in India differ from the prevalence in other continents and countries, and evaluating the reliability of the data.

To the best of our knowledge, this study is the first to document high-risk HPV genotype variations on the Indian subcontinent by using 2 methodologies. However, our results need to be confirmed and updated by studies that use similar, or preferably, identical technology to detect HPV genotypes across all regions and that consistently include all relevant high-risk HPV genotypes. Detailed reporting of quality assurance procedures must also be considered, as well as advances in molecular diagnostic techniques, such as the Xpert HPV assay (Cepheid Inc).^31-35^ The Xpert HPV assay detects 14 high-risk HPV genotypes using a real-time PCR method targeting an 80 to 150 base pair segment in the E6/E7 region and could be used as a point-of-care testing method.^35^

We also determined that variations in prevalence of high-risk HPV genotypes differed from other continents and countries and within different regions/states in India, but the data must be interpreted with caution because, in addition to the deficiencies in the studies included in the scoping review of the literature, the WHO region data and the Indian subcontinent scoping review data are from different periods.

The question of whether regional variations warrant different high-risk HPV vaccine compositions and/or vaccination strategies in different regions in India must be answered in the negative, pending future state-of-the-art investigations that yield more reliable data. Data from both the HPV Information Centre and the scoping review of the literature show that the most prevalent HPV genotypes in India are HPV 16 and HPV 18, and these 2 genotypes are included in the currently available vaccines in India. Although our data show that the remaining 10 high-risk HPV genotypes are present in different regions, our data do not support altering the currently available vaccine compositions. The exceptions noted in the states of Rajasthan and Karnataka are interesting but do not warrant changes to the vaccine compositions. Changes to the high-risk HPV vaccine compositions or vaccination strategies must be based on robust data that consistently include testing for all 12 high-risk HPV genotypes.

Despite the data limitations outlined above, the findings from this study can help to inform hypothesis generation and study design focused on the HPV genotypes that cause cervical cancer. Conducting prospective, homogenous, and well-designed studies in India can influence the conduct of similar studies in the sub-Saharan nations and other Global South countries with a high prevalence of cervical cancer. India has a relatively advanced infrastructure compared to other high prevalence cervical cancer regions and can thus contribute to the WHO cervical cancer elimination strategies.

## CONCLUSION

By using 2 methodologic approaches, this study documents regional variations in high-risk HPV genotypes on the Indian subcontinent and across WHO regions and countries and includes 10 HPV genotypes other than HPV 16 and HPV 18. Findings must be interpreted with caution, however; confirmation or refutation will require prospective, high-quality studies. Our results underscore the necessity for quality data collection that consistently includes testing for all 12 high-risk HPV genotypes, uses state-of-the-art detection techniques, and adheres to rigorous quality assurance procedures. One option is a nationwide registry study with strict quality assurance procedures. While the observed differences do not warrant changes to high-risk HPV vaccine composition or vaccination strategies in India or elsewhere, these findings are likely to stimulate further critical discussion and research in the clinical, translational, and scientific communities focused on high-risk HPV, cervical cancer, and other HPV-related malignancies.

## Figures and Tables

**Appendix Figure.**
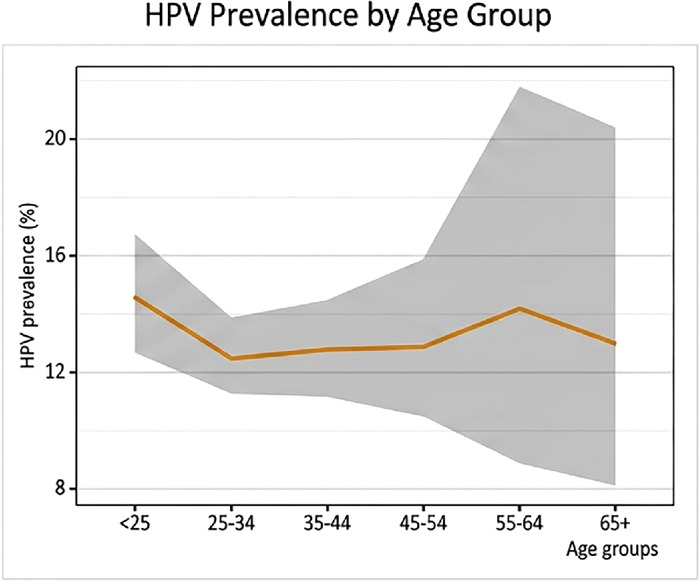
Distribution of human papillomavirus prevalence by age group.

## Notes

*This article meets the Accreditation Council for Graduate Medical Education and the American Board of Medical Specialties Maintenance of Certification competencies for Patient Care, Medical Knowledge, Systems-Based Practice, and Practice-Based Learning and Improvement.*

## Appendix 1. Detailed Search Strategy for the Scoping Review of the Literature

**Table:**

Database	Search Strategy	Number of Results
PubMed	(("human papillomavirus viruses"[MeSH Terms] OR "Human papillomavirus"[Text Word] OR "Human papilloma virus"[Text Word] OR HPV[All Fields]) AND ("genotype"[MeSH Terms] OR Genotype[Text Word] OR Genotypes[Text Word] OR subtype[All Fields] OR subtypes[All Fields] OR variant[All Fields] OR variants[All Fields])) AND ("epidemiology"[MeSH Terms] OR epidemiology[Text Word] OR Distribution[Text Word] OR "genotype distribution" [All Fields] OR "prevalence"[MeSH Terms] OR prevalence[Text Word]) Filters: English, Full Text, Human studies, 2015-2024	2,150
Embase (Elsevier)	(('human papillomavirus viruses'/exp OR 'Human papillomavirus' OR 'Human papilloma virus' OR HPV) AND (genotype/exp OR Genotype OR Genotypes OR subtype OR subtypes OR variant OR variants)) AND (epidemiology/exp OR epidemiology OR Distribution OR 'genotype distribution' OR prevalence/exp OR prevalence)	4,505
Web of Science	((ALL="human papillomavirus viruses" OR ALL="Human papillomavirus" OR ALL="Human papilloma virus" OR ALL=HPV) AND (ALL=genotype OR ALL=Genotype OR ALL=Genotypes OR ALL=subtype OR ALL=subtypes OR ALL=variant OR ALL=variants)) AND (ALL=epidemiology OR ALL=epidemiology OR ALL=Distribution OR ALL="genotype distribution" OR ALL=prevalence OR ALL=prevalence)	2,956
Google Scholar	(Human papilloma virus OR HPV) AND (Genotype OR Subtype OR Variant) AND (Prevalence OR Epidemiology)	86
Total		9,697

## Appendix 2. Studies Excluded After Full-Text Review, n=18

**Table:**

Reason for Exclusion	Citation
Wrong population	Kadian LK, Singhal G, Sharma S, Chauhan P, Nanda S, Yadav R. Incidence and association of HPV16 and 18 with various risk factors in cervical cancer patients in population of Haryana Region, India. *J Clin Diagn Res*. 2019;13(2):QC10-QC13. doi: 10.7860/JCDR/2019/40007.12606
Wrong population	Rawat S, Yadav S, Mandloi P, Panihar C, Barde PV. High incidence of human papillomavirus types 16 and 18 in cervical carcinoma patients in a tertiary care unit, Jabalpur, MP, India. *Indian J Gynecol Oncol*. 2019;17:66:1-7. doi: 10.1007/s40944-019-0307-0
Wrong population	Vijayaraghavan N, Latha KVS, Rahul TS, Kumaravelu S. Prevalence of HPV 16/18 subtypes among invasive cervical cancer patients from a tertiary care hospital in south India: a cross-sectional study. *Indian J Gynecol Oncol*. 2020;18: 105. doi: 10.1007/s40944-020-00456-x
Wrong population	Lall M, Dar L, Bhatla N, et al. Prevalence of human papillomavirus (HPV) genotypes in cervicovaginal secretions of human immunodeficiency virus (HIV) positive Indian women and correlation with clinico-virological parameters. *Front Reprod Health*. 2021;3:695254. doi: 10.3389/frph.2021.695254
Wrong population	Verrier F, Le Coeur S, Delory T. Cervical human papillomavirus infection (HPV) and high oncogenic risk genotypes among women living with HIV in Asia: a meta-analysis. *J Clin Med*. 2021;10(9):1911. doi: 10.3390/jcm10091911
Wrong population	Gupta S, Purwar S, Gupta P, et al. Burden and associated genotype patterns of high-risk human papilloma virus infection and cervical cytology abnormalities among women in central India. *Infect Dis Obstet Gynecol*. 2022;2022:3932110. doi: 10.1155/2022/3932110
Wrong population	Satapathy P, Khatib MN, Neyazi A, et al. Prevalence of human papilloma virus among cervical cancer patients in India: a systematic review and meta-analysis. *Medicine (Baltimore)*. 2024;103(31):e38827. doi: 10.1097/MD.0000000000038827
Wrong study setting	Okoye JO, Chukwukelu CF, Okekpa SI, Ogenyi SI, Onyekachi-Umah IN, Ngokere AA. Racial disparities associated with the prevalence of vaccine and non-vaccine HPV types and multiple HPV infections between Asia and Africa: a systematic review and meta-analysis. *Asian Pac J Cancer Prev*. 2021;22(9):2729-2741. doi: 10.31557/APJCP.2021.22.9.2729
Wrong study setting	Khorsandi N, Vohra P, Samghabadi P, De la Sancha C, Lung D, Long S. Decoding cervical high risk HPV genotype disparities: a comprehensive study on racially, ethnically, and gender diverse populations. *J Am Soc Cytopathol*. 2024;13(5):S29-S30. doi: 10.1016/j.jasc.2024.08.053
Wrong outcome	Kabekkodu SP, Bhat S, Pandey D, et al. Prevalence of human papillomavirus types and phylogenetic analysis of HPV-16 L1 variants from Southern India. *Asian Pac J Cancer Prev*. 2015;16(5):2073-2080. doi: 10.7314/apjcp.2015.16.5.2073
Wrong outcome	Basu P, Muwonge R, Bhatla N, et al. Two-dose recommendation for human papillomavirus vaccine can be extended up to 18 years – updated evidence from Indian follow-up cohort study. *Papillomavirus Res*. 2019;7:75-81. doi: 10.1016/j.pvr.2019.01.004
Wrong outcome	Thakur N, Singhal P, Mehrotra R, Bharadwaj M. Impacts of single nucleotide polymorphisms in three microRNAs (miR-146a, miR-196a2 and miR-499) on the susceptibility to cervical cancer among Indian women. *Biosci Rep*. 2019;39(4):BSR20180723. doi: 10.1042/BSR20180723
Wrong outcome	Hull R, Mbele M, Makhafola T, et al. Cervical cancer in low and middle-income countries. *Oncol Lett*. 2020;20(3):2058-2074. doi: 10.3892/ol.2020.11754
Wrong outcome	Daniels V, Saxena K, Patterson-Lomba O, et al. Modeling the health and economic implications of adopting a 1-dose 9-valent human papillomavirus vaccination regimen in a high-income country setting: an analysis in the United Kingdom. *Vaccine*. 2022;40(14):2173-2183. doi: 10.1016/j.vaccine.2022.02.067
Wrong outcome	Saraiya UB. New paradigms in cervical cancer prevention. *J Obstet Gynaecol India*. 2024;74(4):295-302. doi: 10.1007/s13224-024-02050-z
Duplicate study	Mishra R, Bisht D, Gupta M. Distribution and prevalence of high-risk human papillomavirus infection in women of western Uttar Pradesh, India: a hospital-based study. *JSAFOG*. 2022;14(2):91-94. doi: 10.5005/jp-journals-10006-2013^a^
Small sample size	Shashidhar SC, Sonkusare S, Ramesh PS, Shetty AK, Shetty V, Devegowda D. Prevalence of high-risk human papillomavirus in women with normal and abnormal pap smear: a cross sectional study from a tertiary hospital in South India. *Indian J Gynecol Oncol*. 2021;19(97):1-8. doi: 10.1007/s40944-021-00592-y
Small sample size	Srivastava AN, Misra JS, Rizvi S. Detection of human papillomavirus high-risk genotypes in rural women of Lucknow, North India. *J Cancer Res Ther*. 2021;17(6):1468-1472. doi: 10.4103/jcrt.JCRT_631_19

^a^Although this study has a different title and authors, it is a duplicate of the Punia and Sharma study (2024).^30^ We included the most recently published study in our review and excluded the earlier study.
